# Efficacy and safety of setipiprant in seasonal allergic rhinitis: results from Phase 2 and Phase 3 randomized, double-blind, placebo- and active-referenced studies

**DOI:** 10.1186/s13223-017-0183-z

**Published:** 2017-04-04

**Authors:** Paul Ratner, Charles P. Andrews, Frank C. Hampel, Bruce Martin, Dale E. Mohar, Denis Bourrelly, Parisa Danaietash, Sara Mangialaio, Jasper Dingemanse, Abdel Hmissi, Jay van Bavel

**Affiliations:** 1 Sylvana Research Associates, 7711 Louis Pasteur Drive, Suite 406, San Antonio, TX 78229 USA; 2 Diagnostics Research Group, San Antonio, TX USA; 3 Central Texas Health Research, New Braunfels, TX USA; 4 Southwest Allergy and Asthma Center, San Antonio, TX USA; 5 Kerrville Research Associates, Kerville, TX USA; 6 Actelion Pharmaceuticals Ltd, Allschwil, Switzerland; 7 Isis Clinical Research, Austin, TX USA

**Keywords:** Allergy, Rhinitis, Efficacy, Safety, Mountain Cedar pollen, Setipiprant

## Abstract

**Background:**

Antagonism of chemoattractant receptor-homologous molecule on T-helper type-2 cells (CRTH2), a G-protein coupled receptor for prostaglandin D2, could be beneficial for treating allergic disorders. We present findings on the efficacy and safety/tolerability of a CRTH2 antagonist (setipiprant) in participants with seasonal allergic rhinitis (AR) in a real-life setting over 2 weeks.

**Methods:**

A Phase 2 trial and a Phase 3 trial were conducted at seven centers in Texas, USA during the Mountain Cedar pollen season. Both were prospective, randomized, double-blind, placebo- and active-referenced (cetirizine) studies. The Phase 2 trial assessed setipiprant 100–1000 mg b.i.d. and 1000 mg o.d. versus placebo in adult and elderly participants. The Phase 3 trial assessed setipiprant 1000 mg b.i.d. in adolescent, adult, and elderly participants. Efficacy was assessed using daytime nasal symptom scores (DNSS), night-time nasal symptom scores (NNSS) and daytime eye symptom scores (DESS).

**Results:**

579 participants were randomized in the Phase 2 trial (mean age 41.6–43.4 years); 630 were randomized in the Phase 3 trial (mean age 37.5–40.7 years). A statistically significant, dose-related improvement in mean change from baseline DNSS was observed over 2 weeks with setipiprant 1000 mg b.i.d. versus placebo in the Phase 2 trial (−0.15 [95% CI −0.29, −0.01]; p = 0.030). Setipiprant 1000 mg b.i.d. had no significant effect on this endpoint in the Phase 3 trial (−0.02 [95% CI −0.12, 0.07]; p = 0.652). Total and individual NNSS and DESS symptom scores were significantly improved with setipiprant 1000 mg b.i.d. versus placebo in the Phase 2 but not the Phase 3 trial. Setipiprant showed a favorable safety/tolerability profile.

**Conclusions:**

The Phase 2 trial was the first large clinical study to assess a CRTH2 antagonist in seasonal AR in a real-life setting. Setipiprant dose-related efficacy in the Phase 2 trial was not confirmed during Phase 3. Setipiprant was well tolerated in both studies.

*Trial registration* NCT01241214 and NCT01484119

**Electronic supplementary material:**

The online version of this article (doi:10.1186/s13223-017-0183-z) contains supplementary material, which is available to authorized users.

## Background

Allergic rhinitis (AR) resulting from immunological responses to allergens such as pollen and house dust mites is a very common condition affecting up to 30% of people worldwide [1, 2] _ENREF_1. The early-phase response of AR is associated with rapid-onset symptoms such as sneezing and itchy eyes, while the late-phase inflammatory response is associated with more systemic symptoms such as tiredness. Many sufferers report an impact of AR on daily activities, and health-related quality of life has been shown to correlate negatively with AR severity [3–7]. ENREF_7 A large proportion of affected individuals actively seek new treatments and frequently switch therapies [8].

Several drug classes are available for the treatment of AR, including oral and intra-nasal anti-histamines, intranasal corticosteroids, leukotriene receptor antagonists, and cromolyn sodium [9]. Currently available pharmacotherapies for AR have not yet been shown to provide consistent relief from the full spectrum of clinical symptoms in all of those affected, particularly symptoms affecting the eye and those related to underlying inflammation [2, 10]. While intranasal glucocorticoids and oral or intranasal anti-histamines are generally effective in people with mild-to-moderate AR, they are less effective in those with perennial AR [10, 11]. In addition, some treatment options have side effects that may limit their long-term use, such as epistaxis and possible, rare consequences of systemic exposure with intranasal glucocorticoids [12, 13]. The limitations associated with currently available treatments have fueled continued research into newer and better ways of modulating the underlying immune pathways of AR [14].

Prostaglandin D2 (PGD_2_) is a pro-inflammatory mediator that is considered to affect the inflammatory cascade underlying AR through two cell surface receptors: d-prostanoid type 1 or PGD_2_ receptor type 1 (DP1 or PTGDR1), and chemoattractant receptor-homologous molecule on T-helper type-2 cells (CRTH2, also known as DP2 or PTGDR2) [15–17]. Binding of PGD_2_ to CRTH2, which is expressed on key effector cells in the allergic response cascade, including Th2 cells, eosinophils, group 2 innate lymphoid cells, and basophils, triggers a series of humoral and cellular immune reactions such as chemotactic cellular recruitment, degranulation of eosinophils and basophils, and secretion of interleukins. Together, these actions result in eosinophilia, tissue damage, and tissue remodeling [18–20].

Antagonism of CRTH2 has been suggested as a strategy to counteract the pathophysiological effects of PGD_2_, thereby modulating the symptoms of allergic inflammation [16, 17, 21–23]. This has led to considerable interest and research on the clinical use of CRTH2 antagonists as a novel treatment approach in chronic allergic inflammatory conditions such as AR, allergic dermatitis, and asthma [14, 16, 17, 23–26]. A number of lines of experimental data suggest that CRTH2 antagonists can selectively counteract the pro-inflammatory effects of PGD_2_, which are thought to underlie the allergic response in AR [16, 17, 21, 27–32].

Setipiprant is an orally active tetrahydropyridoindole derivative and a selective CRTH2 antagonist that has been shown to have greater specificity for CRTH2 than for DP1. Preclinical studies have shown that setipiprant blocks the activation of eosinophils and basophils, and reduces the secretion of cytokines (IL-4, IL-5, and IL-13) by Th2 cells [33]. In clinical studies, setipiprant has been shown to be well tolerated at both single and multiple doses in healthy subjects [27, 34–36]. A multicenter, double-blind, placebo-controlled, proof-of-mechanism Phase 2 study in 18 adult male participants with mild allergic asthma (forced expiratory volume in 1 s [FEV_1_] ≥70% predicted) demonstrated that setipiprant 1000 mg b.i.d. was well tolerated and protected against allergen-induced AR and airway hyper-responsiveness to house dust mite extract over a 5-day period [37].

Here, we report results from a Phase 2 trial and a Phase 3 trial that assessed the efficacy, safety, and tolerability of setipiprant in adolescent, adult, and elderly participants with seasonal AR associated with Mountain Cedar (*Juniperus sabinoides*) pollen over a 2-week period.

## Methods

### Design and treatment

Two prospective, randomized, double-blind, placebo- and active-referenced, parallel-group clinical trials evaluated setipiprant in participants with seasonal AR at seven centers in Texas, USA during the Mountain Cedar pollen season. The Phase 2 trial (ClinTrials.gov reference: NCT01241214) was a six-arm dose-ranging trial in adult/elderly male and female participants aged 18–70 years, randomized 1:1:1:1:1:1 to receive placebo, setipiprant at doses of 100 mg b.i.d., 500 mg b.i.d., 1000 mg b.i.d. or 1000 mg o.d., or cetirizine 10 mg o.d. (active reference). The Phase 3 trial (ClinTrials.gov reference: NCT01484119) was conducted in adolescent, adult, and elderly participants (aged 12–76 years, inclusive) randomized 1:1:1 to receive placebo, setipiprant 1000 mg b.i.d., or cetirizine 10 mg o.d. (active reference).

Both trials comprised a 4-week screening period, a 1-week single-blind placebo run-in, a 2-week double-blind randomized treatment period, a 3-day single-blind placebo run-out, and a 30-day safety follow-up. Screened individuals who were eligible to enter the trials started the placebo run-in phase only when the Mountain Cedar season was confirmed as ongoing (i.e., when pollen counts were ≥50 grains/m^3^ for 3 consecutive days). After placebo run-in, participants were re-assessed for eligibility prior to randomization. Participants were required to demonstrate ≥80% compliance in the completion of their run-in diaries and in self-administered run-in medication in order to be enrolled.

To be eligible for randomization after placebo run-in, participants were required to be symptomatic for AR and to have an evening reflective total nasal symptom score (rTNSS) of ≥42/84 over the whole 1-week run-in period, or ≥6/12 on each of 4 consecutive days during the run-in period. The rTNSS was the sum of four individual scores for ‘nasal congestion’, ‘rhinorrhea’, ‘nasal pruritus’, and ‘sneezing’, each scored on a scale ranging from 0 (no symptom) to 3 (severe symptom) inclusive, with a total score range of 0–12 per day. Reflective scores (i.e., symptom severity over 12 h since last placebo dose) were assessed as opposed to instantaneous scores (i.e., symptom severity at the moment of assessment).

In both trials, treatments were allocated using a centralized randomization procedure based on an interactive response system. All study staff, participants, and sponsor personnel were blinded to study treatment up to completion of the studies. In the Phase 2 trial, study medication comprised four capsules (setipiprant, cetirizine, and/or placebo matching both setipiprant and cetirizine) for each of the morning and evening doses, and in the Phase 3 trial, treatments were administered as two tablets (setipiprant or matching placebo) and one capsule (cetirizine or matching placebo) for each of the morning and evening doses. Participants took all study medications 30 min before breakfast in the morning and before going to bed at night.

### Participants

In both trials, all participants had a documented clinical history of seasonal AR associated with Mountain Cedar pollen for the previous 2 years. Prior to enrolment, participants were required to show a positive reaction in a skin-prick test using Mountain Cedar allergen, with a wheal diameter ≥3 mm greater than saline control.

Participants were excluded if they: had non-allergic rhinitis or were receiving other treatments for AR; were receiving treatment for other reasons which could have an impact on AR and/or the primary study endpoint (e.g., systemic steroids); had severe physical nasal obstruction, ongoing chronic respiratory disorders or bacterial/viral infections that could interfere with study assessments; or had asthma requiring treatment other than inhaled short-acting β_2_-agonists.

Written informed consent was obtained from all participants, and all study procedures and materials were reviewed and approved by Institutional Review Boards. Both the Phase 2 study and the Phase 3 study were conducted in accordance with the Declaration of Helsinki and US laws and regulations.

### Pollen count

To document the Mountain Cedar allergy season, pollen counts were measured using a calibrated Rotorod sampler (SDI, Plymouth Meeting, Pennsylvania) every day at one site in each of the five relevant geographic areas containing the seven study centers. The Mountain Cedar pollen season was defined as the time at which the pollen count was ≥50 grains/m^3^ for three consecutive days at each site.

### Concomitant medications

Prohibited concomitant medications included: AR or ocular symptom treatments; nasal irrigation solutions or saline sprays; nasal strips or devices to improve airflow; asthma treatments (except inhaled short-acting β2-agonists); topical calcineurin inhibitors; allergen immunotherapy; systemic immunosuppressive or immunomodulatory treatments; dermatologic corticosteroids (except ≤1% hydrocortisone); insomnia medications; non-steroidal anti-inflammatory drugs; selective cyclooxygenase (COX) inhibitors; and live vaccines; any other investigational drug; or chronic use of intranasal medication (e.g., calcitonin salmon).

### Efficacy endpoints

All participants in the Phase 2 and Phase 3 trials recorded their nasal and eye symptoms daily using electronic diaries with automated symptom questionnaires evaluating the daytime nasal symptom score (DNSS; mean of individual symptom scores for ‘nasal congestion’, ‘rhinorrhea’, ‘nasal pruritus’, and ‘sneezing’), the night-time nasal symptom score (NNSS; mean of ‘difficulty going to sleep’, ‘night-time awakenings’, and ‘nasal congestion’ scores) [38], and the daytime eye symptom score (DESS; mean of ‘tearing’, ‘itching’, ‘redness’, and ‘puffy eyes’ scores) [38]. Individual symptoms comprising each parameter were evaluated on four-point rating scales ranging from 0 (no symptom) to 3 (severe symptom). For each of these parameters, reflective scores represented assessments of symptom severity during the 12 h since the previous dose, and instantaneous scores represented symptoms present at the moment of assessment. For instance, the DNSS was measured as both an evening reflective score (rDNSS) and instantaneous scores just before morning (a.m. iDNSS) and evening doses (p.m. iDNSS).

The primary endpoint in both trials was the mean change from baseline in evening rDNSS over 2 weeks of double-blind treatment, and was tested as the primary analysis comparing setipiprant 1000 mg b.i.d. to placebo. Exploratory assessments of changes in rDNSS with other setipiprant doses were also conducted in the Phase 2 dose-ranging trial.

A number of secondary nasal and ocular symptom endpoints were used to further assess the efficacy of setipiprant versus placebo over the 2-week double-blind treatment period in both studies, including morning reflective NNSS (recorded each day before morning doses) and evening reflective DESS (DESS; recorded each day before evening doses). During the Phase 3 trial, evening reflective daytime nasal congestion score (rDNCS; the individual ‘nasal congestion’ item from the DNSS) was specifically analyzed to reflect nasal congestion through each day. In addition, morning reflective TNSS (rTNSS), and morning and evening instantaneous TNSS (a.m. iTNSS and p.m. iTNSS, respectively) were assessed throughout Phase 3 double-blind treatment.

Disease-specific patient quality-of-life (QoL) was assessed at randomization and at the end of weeks 1 and 2 in both trials using the rhinoconjunctivitis quality-of-life questionnaire (RQLQ) [39].

### Pharmacokinetics

Previous clinical studies with setipiprant showed a relationship between trough plasma concentration and AUC_τ_ (i.e., AUC during a dosing interval) [34] indicating that trough plasma setipiprant concentrations allow assessment of systemic drug exposure as well as treatment compliance. Trough plasma setipiprant concentrations were therefore used to assess systemic drug exposure and treatment compliance using a validated liquid chromatography coupled with tandem mass spectrometry (LC-MS/MS) assay, which had a lower limit of quantification (LLQ) of 1 ng/mL [36]. Measurements were performed using 2-mL venous blood samples taken before the morning administration of study medication at the end of weeks 1 and 2 of randomized treatment.

### Tolerability and safety

Treatment-emergent adverse events (AEs) and serious adverse events (SAEs) were recorded throughout both trials. Physical examinations and clinical laboratory tests were performed at screening and at the end of the treatment: all laboratory assessments were performed at a certified centralized laboratory (ACM Global Central Laboratory, USA). Vital signs and 12-lead electrocardiogram (ECGs; centrally read at ERT, Philadelphia PA, USA) were performed at screening, randomization, and at the end of double-blind treatment.

### Data analysis

Statistical analyses in both the Phase 2 and Phase 3 trials addressed the primary null hypothesis that there was no difference between setipiprant 1000 mg b.i.d. and placebo with respect to the mean change from baseline in rDNSS over the 2-week double-blind treatment period. In the Phase 2 trial, it was expected that 86 evaluable participants per treatment arm would provide 90% power to detect a difference of −0.25 in DNSS with setipiprant 1000 mg b.i.d. vs placebo if the change from baseline in DNSS in both groups was normally distributed with a standard deviation (SD) of 0.50. With the assumption that 5% of treated participants may not be evaluable for the primary analysis, the total number of participants to be randomized into the six treatment arms was set at 546. For the Phase 3 trial, assuming that the mean change from baseline over a 2-week treatment period in DNSS in both setipiprant and placebo groups was normally distributed with a common SD of 0.55, 198 participants per arm provided 95% power to detect a clinically relevant difference of 0.20 between the setipiprant and placebo groups, with a significance level (alpha) of 0.05 using a two-sided two-sample t test. With the assumption that approximately 5% of the participants randomized would be not evaluable or not treated, the total number of participants to be randomized into the three treatment arms was set at 630 (210 per group).

No correction for multiplicity was applied in the Phase 2 trial, while the null hypothesis was tested in a fixed sequence in the Phase 3 trial to account for multiplicity across the efficacy endpoints, and to control the two-sided type-I error rate at the 5% significance level (sequence: NNSS, rDNCS, DESS, rTNSS, p.m. iTNSS, a.m. iTNSS).

An evaluation of the dose–response relationship based on rDNSS across Phase 2 b.i.d. setipiprant dosing groups was conducted using a modification of the MCP-Mod method using the dose finding procedure of R v2.11.1 software [40, 41]. Candidate parametric models were investigated using multiple-comparison goodness of fit techniques to identify the model that best represented the observed dose–response relationship.

Analyses of Phase 2 and Phase 3 secondary efficacy endpoints were exploratory in nature, with efficacy summarized by absolute changes from baseline (mean ± SD and/or least-squares mean ± standard error [SE], median and range), and mean (95% CI) differences between setipiprant and placebo groups. The main efficacy analyses were based on the modified all-treated data set (all randomized participants who took ≥1 dose of the double-blind study medication and had baseline and ≥1 post-baseline efficacy assessment) in the Phase 2 trial, and on the modified intent-to-treat population (mITT; all randomized participants who received at least one dose of double-blind study medication) in the Phase 3 trial. Sensitivity analyses conducted in both trials included evaluations based on the per-protocol population (PP set; all randomized, eligible participants who completed the trials without major protocol deviations) and supportive analyses of covariance (ANCOVA).

For pharmacokinetic analyses, trough plasma setipiprant concentrations were summarized using arithmetic and geometric mean ± SD and median (range). Tolerability and safety, assessed in both studies based on the respective study safety sets (all randomized participants who received at least one dose of study medication) were evaluated using descriptive categorical data (n [%]). Pollen counts were recorded as the mean ± SD of daily counts (per m^3^ of air) over the single-blind run-in and 2-weeks treatment periods.

## Results

### Participants

In the Phase 2 trial, 736 out of 849 participants screened entered the run-in phase, and 579 were randomized (96–98 participants per treatment group) (Additional file 1: Figure S1a). Overall, 557 participants (96.2%) completed the trial and 22 (3.8%) discontinued prematurely. The most frequent reasons for premature discontinuation were withdrawal of consent (n = 11) and administrative reasons (n = 10).

In the Phase 3 trial 832 of the 930 participants screened entered the run-in phase, from whom 630 were randomized (210 per treatment group) (Additional file 1: Figure S1b). A total of 604 participants completed the trial, and a total of 19 (3.0%) discontinued prematurely. The most frequent reasons for premature discontinuation were ‘patient’s decision’ (n = 7) and ‘adverse events’ (n = 6).

Demographics and baseline disease characteristics were generally balanced across the treatment groups in both the Phase 2 trial (Table 1) and the Phase 3 trial (Table 2). The mean ages across treatment groups in the Phase 2 trial ranged from 41.6 to 43.4 years, and the mean duration over which participants had experienced AR was similar across the treatment groups (18.8–21.7 years). Mean ages across treatment groups were lower in the Phase 3 trial (37.5–40.7 years; 6.2–10.5% per group aged >12 and <18 years). The mean duration of AR ranged from 18.8 to 21.7 years across treatment groups in the Phase 2 trial, and from 17.4 to 18.9 years across the Phase 3 treatment groups. Overall, mean baseline DNSS scores were similar across treatment groups and between the two studies: means per treatment group ranged from 2.24 to 2.26 in the Phase 2 trial and from 2.16 to 2.22 in the Phase 3 trial.

**Table 1:** Patient demographics and baseline disease characteristics (Phase 2 trial)

	Placebo	Setipiprant 100 mg b.i.d.	Setipiprant 500 mg b.i.d.	Setipiprant 1000 mg b.i.d.	Setipiprant 1000 mg o.d.	Cetirizine 10 mg o.d.
Patients, n	96	98	97	96	96	96
Age in years, mean (SD)	42.2 (13.0)	43.0 (12.1)	42.0 (13.9)	41.6 (11.7)	41.8 (12.8)	43.4 (12.8)
Sex (% male: % female)	28: 72	24: 77	32: 68	30: 70	35: 65	30: 70
Ethnicity, n (%):						
Caucasian	62 (65)	56 (57)	56 (58)	58 (60)	47 (49)	51 (53)
Black	3 (3)	9 (9)	9 (9)	7 (7)	7 (7)	6 (6)
Hispanic	31 (32)	30 (31)	32 (33)	31 (32)	41 (43)	39 (41)
Other	–	3 (3)	–	–	1 (1)	–
AR duration in years, mean (SD)	18.8 (12.2)	19.3 (11.4)	19.1 (11.5)	18.9 (12.3)	19.8 (12.3)	21.7 (16.8)
Baseline DNSS, mean (SD)^a^	2.25 (0.42)	2.25 (0.40)	2.26 (0.43)	2.26 (0.41)	2.24 (0.41)	2.24 (0.42)

All-randomised set

*AR* allergic rhinitis, *DNSS* daytime nasal symptom score, *SD* standard deviation

^a^Modified all-treated set

**Table 2:** Patient demographics and baseline disease characteristics (Phase 3 trial)^1^

	Placebo	Setipiprant 1000 mg o.d.	Cetirizine 10 mg o.d.
Patients, n	210	210	210
Age in years, mean (SD)	37.5 (13.9)	38.2 (15.1)	40.7 (14.2)
Sex, (male: female)	40%: 60%	36%: 64%	39%: 61%
Ethnicity, n (%):			
Caucasian	107 (51)	102 (49)	98 (47)
Black	12 (6)	13 (6)	20 (10)
Hispanic	90 (43)	92 (44)	89 (42)
Other	1 (<1)	3 (<2)	3 (<2)
AR duration in years, mean (SD)	17.4 (11.9)	18.6 (11.5)	18.9 (11.5)
Baseline DNSS, mean (SD)^a^	2.16 (0.43)	2.17 (0.41)	2.22 (0.42)

All-randomised set

*AR* allergic rhinitis, *DNSS* daytime nasal symptom score, *SD* standard deviation

^a^ITT (intent-to-treat) set

**Fig. 1:**
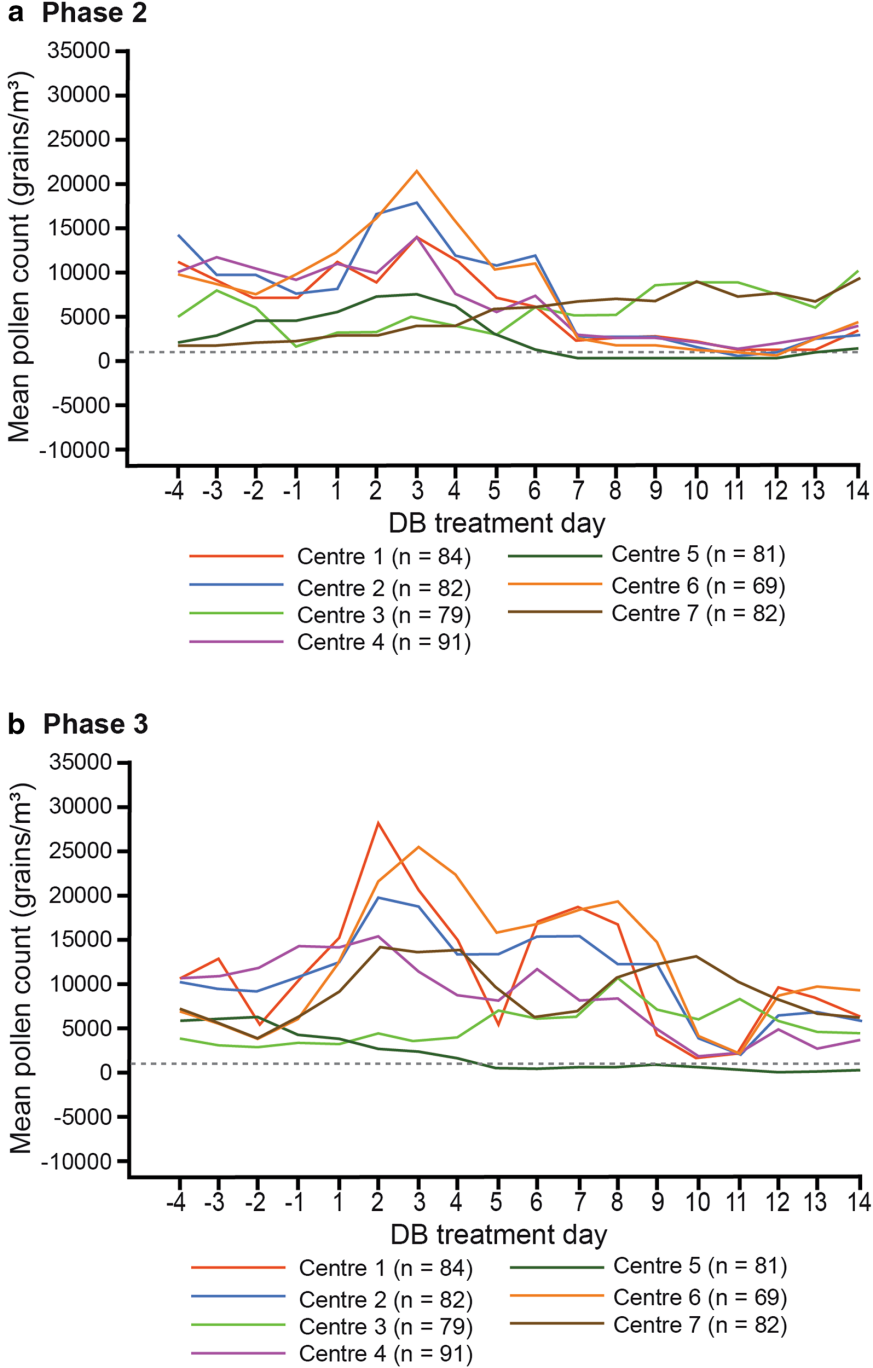
Exposure to Mountain Cedar allergen by day at each center prior to and during **a** Phase 2 and **b** Phase 3 trials. Data points are means of multiple measurements per center

Mean pollen counts at each study region throughout the studies are shown in Fig. 1. Participants in both studies were exposed to sufficient pollen allergen during the course of the study to allow appropriate assessment of AR profile and treatment effects (see also Additional file 2: Figure S2 for overall mean pollen counts across centers throughout each study).

### Primary efficacy

A significant treatment effect of setipiprant 1000 mg b.i.d. versus placebo on the primary efficacy endpoint (mean absolute change from baseline in rDNSS over 2 weeks) was observed in the Phase 2 trial (−0.15 [95% CI −0.29, −0.01]; p = 0.030) but not in the Phase 3 trial (−0.02 [95% CI −0.12, 0.07]; p = 0.652). In contrast, statistically significant treatment effects were observed in both studies with the active reference, cetirizine 10 mg o.d.: −0.21 (95% CI −0.35, −0.07; p < 0.001 vs. placebo) in the Phase 2 trial and −0.23 (95% CI −0.32, −0.13; p < 0.001 vs. placebo) in the Phase 3 trial. These findings were supported in both studies by additional sensitivity analyses (PP set and ANCOVA on modified all-treated set [Phase 2] or mITT set [Phase 3]).

**Fig. 2:**
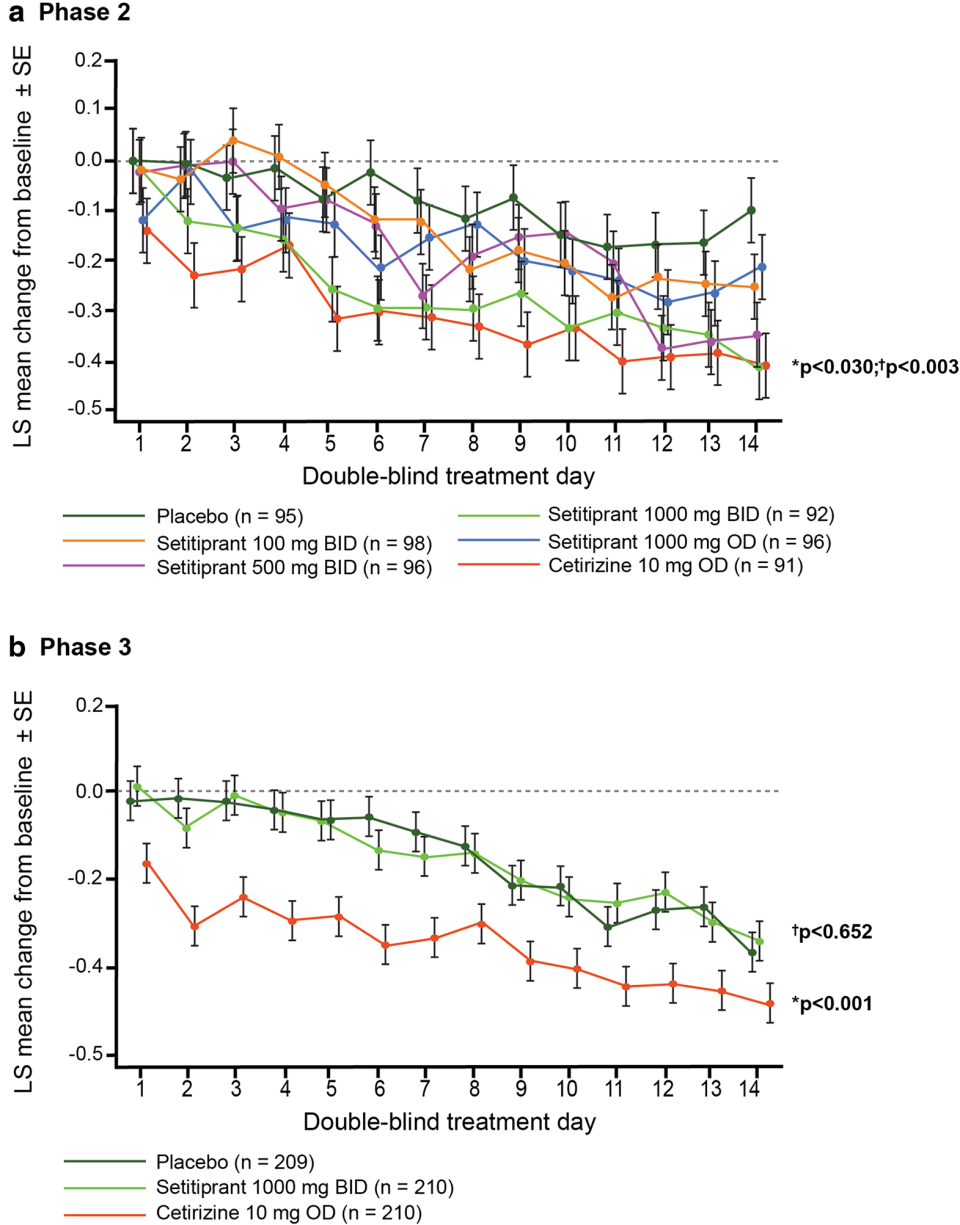
Changes in rDNSS per day over the 2-week randomized treatment period. **a** Phase 2 data based on modified all-treated set) and **b** Phase 3 data based on modified ITT (intent-to-treat) set. Data are mean ± standard error (SE). *p values for mean changes from baseline with setipiprant vs. placebo; ^†^p values for mean changes from baseline with cetirizine vs. placebo

During the Phase 2 trial there was a clear separation in changes in rDNSS from baseline between the setipiprant 1000 mg b.i.d. group and placebo values from day 2 onwards (Fig. 2a). Cetirizine was associated with the greatest treatment effect versus placebo throughout double-blind treatment. During the Phase 3 trial, mean (SE) changes from baseline in rDNSS by day did not indicate sufficient separation between setipiprant 1000 mg b.i.d. and placebo to define any treatment effect (Fig. 2b). There was a clear treatment effect compared with placebo throughout the double-blind treatment period in the cetirizine active control group.

### Secondary efficacy

**Fig. 3:**
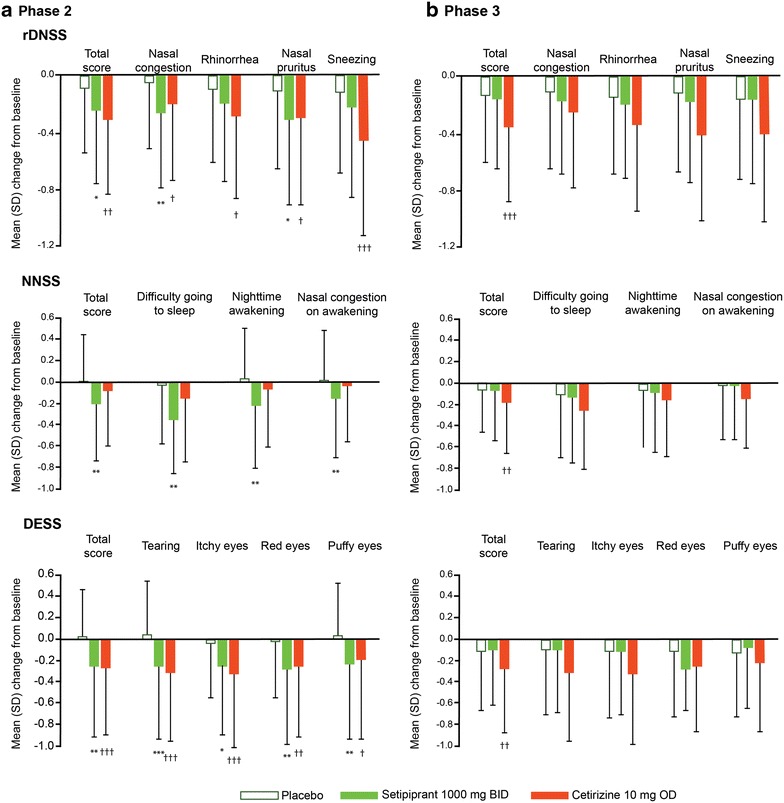
Changes from baseline in individual items from the reflective DNSS, NNSS, and DESS after 2 weeks of randomized therapy. **a** Phase 2 trial (based on modified all-treated set) and **b** Phase 3 trial based on modified ITT (intent-to-treat) set. All data are expressed as mean (95% CI). *DESS* reflective daytime eye symptom score, *rDNSS* reflective daytime nasal symptom score, *NNSS* night-time nasal symptoms score, *RQLQ* rhinoconjunctivitis quality-of-life questionnaire. *p < 0.05; **p < 0.01; ***p < 0.001 for change from baseline with setipiprant vs. placebo. ^†^p < 0.05; ^††^p < 0.01; ^†††^p < 0.001 for change from baseline with cetirizine vs. placebo

Mean changes from baseline in both total and individual symptom scores from the rDNSS, NNSS, and DESS after 2 weeks are summarized for both trials in Fig. 3.

The largest mean differences in individual rDNSS symptom scores with setipiprant 1000 mg b.i.d. versus placebo during the Phase 2 trial were observed for ‘nasal congestion’ (−0.21 [95% CI −0.36, −0.07]; p = 0.003) and ‘nasal pruritus’ (−0.20 [95% CI −0.37, −0.03]; p = 0.019) (Fig. 3a). Treatment differences in the other setipiprant dose groups ranged from −0.05 to −0.10: a statistically significant improvement in ‘nasal congestion’ was observed with setipiprant 1000 mg o.d. (p = 0.037). Significant treatment differences versus placebo were observed with cetirizine for all rDNSS symptom scores (p values ranged from 0.03 to <0.001). In the Phase 3 trial, setipiprant 1000 mg b.i.d. was not associated with significant treatment differences versus placebo for any of the individual rDNSS symptom scores (Fig. 3b). In the cetirizine group, treatment differences versus placebo in individual rDNSS symptom scores ranged from −0.29 to −0.14.

Significant mean improvements in the overall NNSS (p = 0.004) and in individual scores for ‘difficulty in going to sleep’ (p = 0.008), ‘night-time awakenings’ (p = 0.004), and ‘nasal congestion on awakening’ (p = 0.025) were observed with setipiprant 1000 mg b.i.d. versus placebo in the Phase 2 trial, (Fig. 3a). Smaller mean improvements were observed in the other setipiprant dose groups, ranging from −0.02 to −0.15. With cetirizine, improvements in NNSS were notably smaller than those seen with setipiprant 1000 mg b.i.d, and were not significant versus placebo. However, in the Phase 3 trial, no significant improvements were observed in either total NNSS or individual NNSS symptom scores with setipiprant 1000 mg b.i.d. versus placebo (Fig. 3b), while a statistically and clinically significant treatment difference was observed with cetirizine (p = 0.012).

Significant mean improvements in total DESS (p = 0.002) and all individual symptom scores for ‘tearing’ (p < 0.001), ‘itchy eyes’ (p = 0.017), ‘red eyes’ (p = 0.003), and ‘puffy eyes’ (p = 0.005) were observed over 2 weeks with setipiprant 1000 mg b.i.d. versus placebo in the Phase 2 trial (Fig. 3a). Mean changes in the other setipiprant dose groups were smaller, ranging from −0.06 to −0.21 across all total and individual symptom scores. In the cetirizine group, significant mean improvements in both total and individual DESS scores were observed (p values ranged from 0.02 to <0.001) that were similar to those observed with setipiprant 1000 mg b.i.d. In the Phase 3 trial, there were no significant improvements in DESS with setipiprant 1000 mg b.i.d. versus placebo (Fig. 3b). In contrast, significant mean improvements in total DESS and individual DESS symptom scores were observed in the cetirizine group that were consistent with those seen with cetirizine in the Phase 2 trial.

**Fig. 4:**
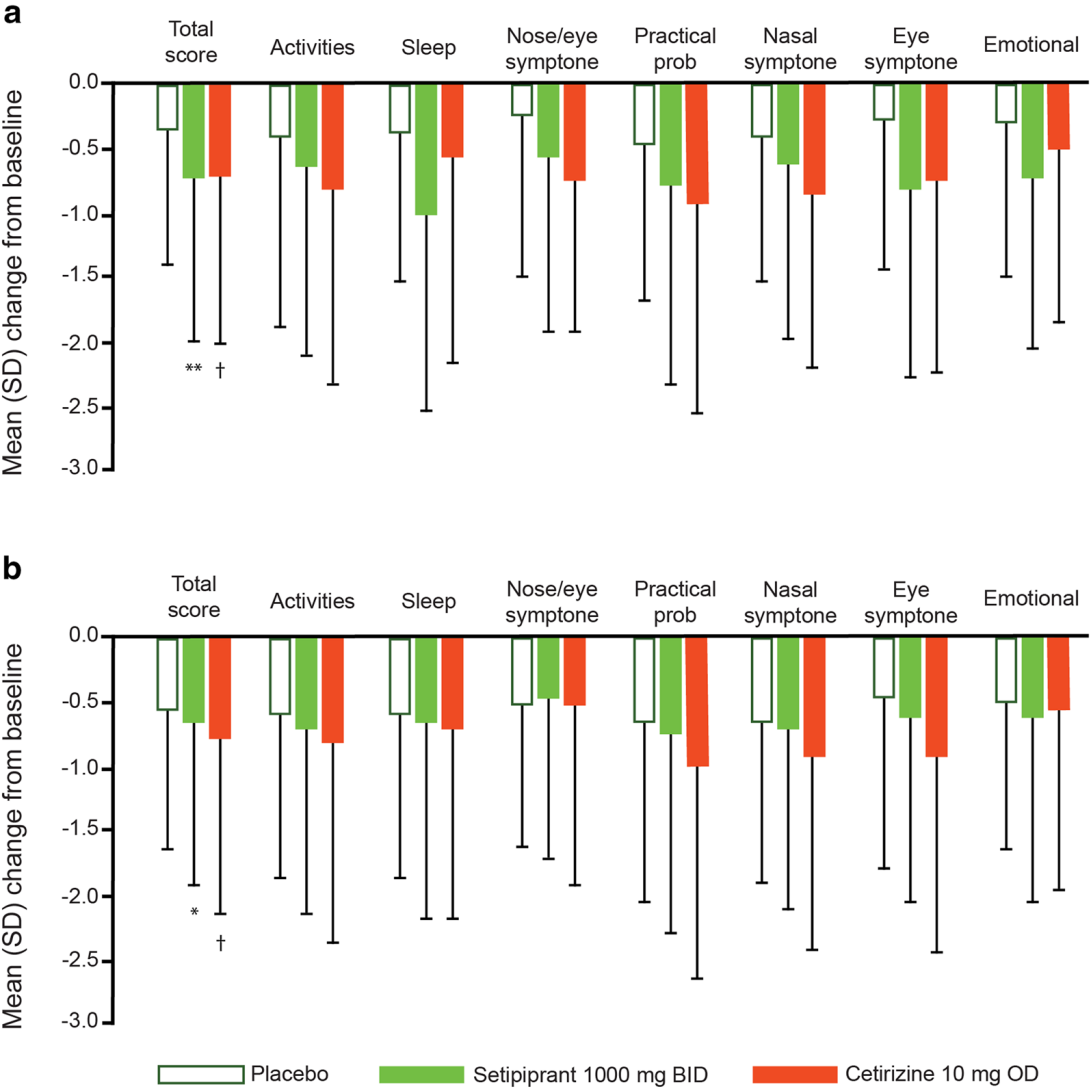
Changes from baseline in total and individual item scores of the RQLQ after 2 weeks of randomized therapy. **a** Phase 2 trial (based on modified all-treated set) and **b** Phase 3 trial based on modified ITT (intent-to-treat) set. All data are expressed as mean (95% CI). *RQLQ* rhinoconjunctivitis quality-of-life questionnaire. *p < 0.05 for change from baseline with setipiprant vs. placebo; ^†^p < 0.05 for change from baseline with cetirizine vs. placebo

Overall RQLQ scores improved significantly with setipiprant 1000 mg b.i.d. at the end of both weeks 1 (p = 0.005) and 2 (p = 0.026) in the Phase 2 trial (Fig. 4a). Mean changes were smaller among the other setipiprant dose groups, ranging from −0.01 to −0.19. Significant improvements were also observed with cetirizine at both time points (p = 0.0253 and p = 0.050). In the Phase 3 trial, no significant improvements in RQLQ were observed with setipiprant 1000 mg b.i.d. after either study week (Fig. 4b), while significant improvements were observed with cetirizine, in line with Phase 2 findings.

No significant or clinically relevant improvements in secondary Phase 3 study endpoints (rDNCS, rTNSS, a.m. iTNSS, and p.m. iTNSS) were observed with setipiprant 1000 mg b.i.d. versus placebo over 2 weeks. In contrast, significant mean treatment effects versus placebo were observed for each of these endpoints with cetirizine (p < 0.001 in all cases).

### Dose–response analysis

**Fig. 5:**
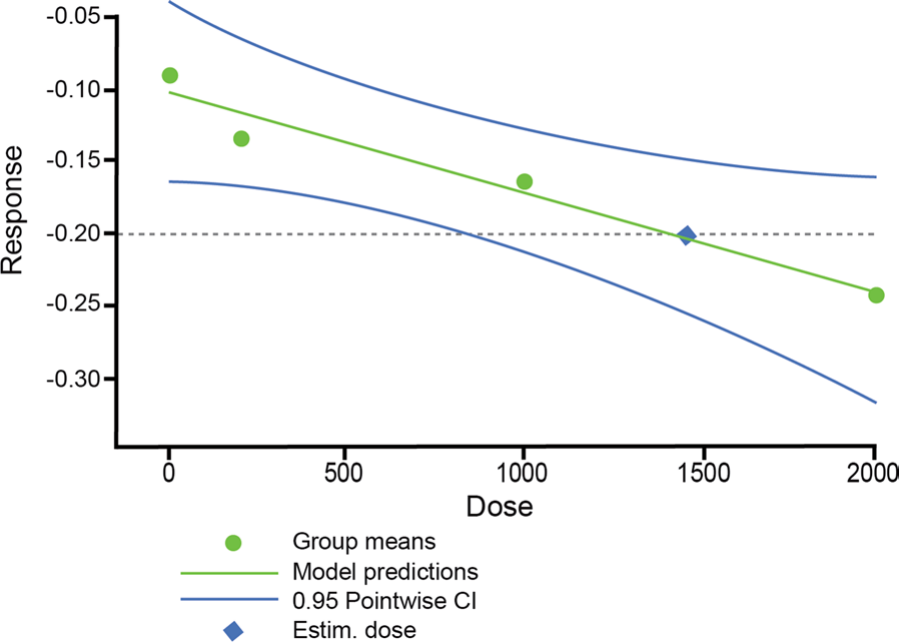
Linear relationship between setipiprant daily dose and change from baseline in rDNSS during Phase 2 dose-ranging trial. *Black dots* represent the observed response (change from baseline in rDNSS) for each dose assessed; *solid black line* represents the dose–response model selected from multiple comparison goodness-of-fit modeling; *blue lines* represent 95% confidence limits, and the *blue symbol* is the calculated dose providing minimally clinically relevant effect (*dashed horizontal line*)

Investigation of several candidate parametric models using multiple comparison techniques showed that a linear model best represented the dose-response relationship for changes in rDNSS across the setipiprant 100–1000 mg b.i.d. dose groups (Fig. 5). Analyses based on the selected linear model identified a total daily setipiprant dose of 1500 mg as providing a clinically relevant effect on rDNSS (−0.2 vs. baseline).

### Pharmacokinetics

Pharmacokinetic analyses across the setipiprant b.i.d. dose groups in the Phase 2 trial indicated a proportional relationship between setipiprant dose and trough setipiprant plasma concentration during both weeks 1 and 2, and no notable differences were observed in setipiprant plasma concentrations between the 2 weeks (Additional file 3: Table S1). Arithmetic mean (95% CI) concentrations in the 100 mg b.i.d., 500 mg b.i.d., and 1000 mg b.i.d. groups in week 2 were 290 (−167, 747), 1299 (−1180, 3779), and 2276 (−1945, 6498) ng/mL, respectively: the mean (95% CI) plasma setipiprant concentration 6–15 h after 1000 mg o.d. (evening) dosing was 1826 (−2252, 5905) ng/mL. In the Phase 3 trial, the mean (95% CI) trough setipiprant plasma concentration at the end of week 2 was 1788 (1405, 2170) ng/mL.

Trough setipiprant plasma concentrations were appreciably higher during both randomized treatment weeks in the setipiprant 1000 mg b.i.d. dose group during the Phase 2 trial (arithmetic mean, 2276–2364 ng/mL; median, 1540–1920 ng/mL) than in the Phase 3 trial (arithmetic mean, 1671–1788 ng/mL, median, 1120 ng/mL in both treatment weeks). Indeed, the mean setipiprant plasma concentration in the 1000 mg b.i.d. group in the Phase 3 trial was between those in the 500 and 1000 mg b.i.d. groups in the Phase 2 trial. The median value in the Phase 3 trial was closer to that observed in the Phase 2 trial 500 mg b.i.d. dose group.

### Safety and tolerability

Overall, there was no clear pattern in the incidence or severity of any adverse event with setipiprant during either the Phase 2 or the Phase 3 trial. The overall incidence of adverse events was greater in the placebo group (13.5%) than in any of the setipiprant dose groups (7.1–9.5%) or in the cetirizine group (6.3%) in the Phase 2 trial. The same was observed in the Phase 3 trial: 16.3% in the placebo group, 11.4% in the setipiprant group, and 11.0% in the cetirizine group. The most frequently reported adverse event in both trials was dry mouth, which occurred in three (3.1%) participants in the setipiprant 500 mg b.i.d. group but not in any other treatment groups in the Phase 2 trial, and in four (1.9%) setipiprant 1000 mg b.i.d.-treated participants, three (1.4%) placebo-treated participants, and two (1.0%) cetirizine-treated participants in the Phase 3 trial.

A total of five participants discontinued study treatment due to AEs in the Phase 2 trial. One participant (1.1%) discontinued setipiprant 1000 mg b.i.d. treatment due to upper respiratory tract infection. In the setipiprant 1000 mg o.d. group, two participants (2.1%) discontinued treatment: one due to upper respiratory tract infection, and the other due to sinusitis. In the cetirizine group, two participants (2.1%) discontinued treatment: one due to sinusitis and the other due to tympanic membrane perforation. Both adverse events were reported as resolved without sequelae on follow-up. There were no discontinuations due to AEs in the other setipiprant groups or the placebo group. AEs leading to discontinuation during the Phase 3 trial were reported in one (0.5%), five (2.4%), and two participants (1.0%) in the setipiprant, placebo, and cetirizine groups, respectively. One participant in the setipiprant group discontinued (did not enter) the single-blind placebo run-out due to cholelithiasis. In the placebo group, increased hepatic enzyme, acute sinusitis, headache, asthma, and insomnia led to discontinuations during double-blind treatment in one participant each. In the cetirizine group, one participant discontinued double-blind treatment due to bronchitis and one participant discontinued the single-blind placebo run-out due to pregnancy.

There were no deaths during either trial, and there were no serious adverse events in the Phase 2 trial. One serious adverse event (cholelithiasis not considered related to study treatment) was recorded in the Phase 3 trial in a participant treated with setipiprant 1000 mg b.i.d. and resulted in discontinuation of treatment. This event, which required hospitalization and cholecystectomy, resolved with clinical sequelae 2 days after the end of the double-blind treatment period.

In both trials, mean changes in hematology variables from baseline were small and unremarkable across all treatment groups. One participant in the setipiprant 100 mg b.i.d. group in the Phase 2 trial had a transient increase in alanine aminotransferase (ALT) activity of >5× upper limit of normal (ULN) approximately 15 days after the end of treatment: this participant’s ALT was >3× ULN at screening and >2× ULN at repeat measurement 21 days before double-blind treatment start. Three participants in the setipiprant group in the Phase 3 trial (two adolescents and one adult) had ALT and/or aspartate aminotransferase (AST) >3× ULN. No participants in the placebo or cetirizine groups had similar findings. Another participant in the setipiprant group had an increase in total bilirubin (from 22.8 μmol/L at screening to 38.5 μmol/L on day 15 [after double-blind treatment was stopped], but total bilirubin fell to 24.2 μmol/L by day 20. These findings were not considered clinically relevant, and were not associated with any AEs. No participants in either trial had increases in ALT or AST >3× ULN concomitant with total bilirubin >2× ULN.

There were no clinically relevant changes in vital signs (mean blood pressure, heart rate, and body weight). No ECG abnormalities were considered clinically relevant and none were reported as adverse events.

## Discussion

The Phase 2 dose-ranging trial presented here is to our knowledge the first large clinical trial to assess a CRTH2 antagonist in a real-life AR setting, and indicated that setipiprant was effective in relieving the symptoms of seasonal AR at a dose of 1000 mg b.i.d. This is in line with the growing clinical evidence from several CRHT2 antagonists investigated in allergen challenge trials supporting the therapeutic role of CRTH2 antagonism in the treatment of AR as evidenced by improvements of nasal and ocular symptoms as well as reduction of inflammatory cells and markers in the nose [25, 28–31, 42–44].

The setipiprant 1000 mg b.i.d. dose was selected for testing in the two trials described here based on findings from a Phase 2 proof-of-mechanism study that demonstrated significant improvements in FEV_1_ versus placebo during the late allergic response [37]. Lower doses (100 mg b.i.d. and 500 mg b.i.d.) were selected for the Phase 2 trial reported here to investigate the effective dose range and to explore the dose–response relationship. The 1000 mg o.d. dose was also tested for comparative purposes, and to address the possible application of a more convenient clinical dosing regimen.

Linear dose-related improvements in the primary efficacy endpoint (mean change in rDNSS over 2 weeks versus placebo) were observed, with significant and clinically relevant improvements in the 1000 mg b.i.d. dose group versus placebo. Good tolerability was also demonstrated over 2 weeks across the setipiprant dose range. Even though the Phase 2 trial was not powered to demonstrate reductions in NNSS or DESS, it is notable that statistically significant improvements in both total and individual symptom scores on these parameters were observed in the setipiprant 1000 mg b.i.d. group. This suggested potential benefits over certain other available treatments for AR (e.g., intranasal corticosteroids) [9].

The encouraging Phase 2 trial findings led to the selection of setipiprant 1000 mg b.i.d. for further clinical development. However, no therapeutic effects were observed for this dose versus placebo in the Phase 3 trial. The primary efficacy analysis in the Phase 3 trial (identical to that in the Phase 2 trial) did not indicate any statistically significant or clinically relevant treatment effect on daytime nasal symptom score with setipiprant 1000 mg b.i.d. versus placebo. Similarly, no treatment effect was observed with setipiprant versus placebo for any of the secondary efficacy endpoints.

Reasons for the apparent difference in efficacy findings for setipiprant 1000 mg b.i.d. between the Phase 2 and Phase 3 trials are not fully known. The two trials had similar study design, adequate statistical power, and were performed in line with guidelines for the development of medicinal products for seasonal AR, with potential sources of bias minimized by the randomized, double-blind nature of the studies [45]. Both trials were conducted in well-defined, balanced populations of participants with a documented history of seasonal AR. While the Phase 3 trial included adolescents in addition to adult and elderly participants, as included in the Phase 2 trial, only 6–11% of participants (per treatment group) in the Phase 3 trial were adolescents, and efficacy findings among these participants were not different to those in adult and elderly participants. All participants in both trials were exposed to adequate pollen counts throughout the treatment periods, at the same centers, to elicit AR symptoms. The same approved active reference drug—the non-sedating antihistamine, cetirizine 10 mg o.d.—was assessed alongside setipiprant in both studies, and showed efficacy consistent with previously reported data [46]. This indicates that the design, setup and degree of pollen exposure were adequate in both studies.

It is noteworthy that the placebo treatment effect was marginally greater in the Phase 3 trial than in the Phase 2 trial: mean changes from baseline after 2 weeks on placebo were −0.13 and −0.09, respectively. However, this difference is not considered sufficient to explain fully the difference in efficacy findings between the two trials, as efficacy findings with the active reference drug, cetirizine, were generally consistent between the two trials.

Another possible factor might be that differences in the pharmacokinetic exposure to setipiprant contributed to the relative lack of observed efficacy in the Phase 3 trial compared with the Phase 2 trial. Assessment of trough setipiprant plasma concentrations at the end of weeks 1 and 2 in the Phase 2 trial indicated that steady-state pharmacokinetics were consistently attained after 1 week of treatment across the dose range assessed, which is in accordance with previous data from healthy subjects [34, 36]. However, both mean and median trough setipiprant concentrations were lower with setipiprant 1000 mg b.i.d. in the Phase 3 trial than in participants receiving the same dose in the Phase 2 trial, and the median value in the Phase 3 trial was closer to that observed in the 500 mg b.i.d. dose group in the Phase 2 trial.

It is also possible that the differences in Phase 2 versus Phase 3 efficacy and pharmacokinetic findings with setipiprant 1000 mg b.i.d. were related to the fact that setipiprant was formulated as 250 mg capsules in the Phase 2 trial, whereas a reformulated version of setipiprant (500 mg tablets) was used in the Phase 3 trial. However, a biocomparison study performed after the drug was reformulated indicated that the pharmacokinetic characteristics of oral setipiprant 500 mg tablets matched those provided by 2 × 250 mg capsules [36]. It is also noteworthy that, in pharmacokinetic–pharmacodynamic modeling studies based on a CRTH2 internalization assay, the median percentage of subjects with at least 90% CRTH2 blockage at trough plasma setipiprant concentrations was higher with 1000 mg b.i.d. than with 500 mg b.i.d. doses, but still remained below 30% [42]. This might also partly explain the apparent discrepancies in clinical efficacy between these two very similar studies.

Finally, based on findings from a pharmacometric/pharmacodynamic modelling study of CRTH2 internalization that was conducted to optimize therapeutic CRTH2 antagonist dose selection, it is possible that the 1000 mg b.i.d. dose of setipiprant provided borderline efficacy. This may also partly explain the discrepancies between Phase 2 and 3 findings.

Overall, setipiprant was well tolerated, and no safety issues were identified during either the Phase 2 or the Phase 3 trial. There was a low overall frequency of treatment-emergent adverse events, and no clinically relevant pattern of abnormal laboratory findings, physical examination findings, vital signs measurements or ECG findings.

## Conclusions

The Phase 2 dose-ranging trial data presented here provided strong evidence of dose-related efficacy with setipiprant in participants with a documented history of AR, but these findings were not confirmed in the subsequent Phase 3 trial. It is notable, however, that setipiprant was well tolerated in both studies. With findings from both trials in hand, the clinical development of setipiprant for the treatment of AR was discontinued.

Clinical trials with more potent CRTH2 antagonists are expected to further elucidate the possible utility of CRTH2 antagonists in this indication [42]. For instance, a recent randomized, double-blind, placebo-controlled clinical study has investigated the single- and multiple-dose tolerability, pharmacokinetics, and pharmacodynamics of a potent new CRTH2 antagonist, ACT-453859, and showed dose-dependent blockage of CRTH2 and good tolerability at doses up to 800 mg o.d. [43]. Further, the pharmacodynamic effect of ACT-453859 after multiple o.d. dosing was maintained over 24 h at levels equal to or greater than those observed 12 h after setipiprant 1000 mg b.i.d. dosing, which suggests that once-a-day dosing could be suitable with this investigational compound.

## Additional files

**Additional file 1: Figure S1.** Patient disposition in a) Phase 2 trial and b) Phase 3 trial.

**Additional file 2: Figure S2.** Overall pollen counts by day prior to and during Phase 2 and Phase 3 trials.

**Additional file 3: Table S1.** Setipiprant trough plasma concentrations across dose groups at the end* of weeks 1 and 2 during Phase 2 and Phase 3 trials.

## Abbreviations

AEs: adverse events
ALT: alanine aminotransferase
ANCOVA: analyses of covariance
AR: allergic rhinitis
AST: aspartate aminotransferase
AUC: area under the curve
CI: confidence interval
CRTH2: chemoattractant receptor-homologous molecule on T-helper type-2 cells
DESS: daytime eye symptom score
DNCS: daytime nasal congestion score
DNSS: daytime nasal symptom score
DP1/2: d-prostanoid type ½
ECGs: electrocardiogram
FEV_1_: forced expiratory volume in 1 s
IL-4/IL-5/IL-13: interleukin 4/5/13
LC-MS/MS: tandem mass spectrometry
LLQ: lower limit of quantification
NNSS: night-time nasal symptom score
PGD_2_: prostaglandin D2
PTGDR1/2: PGD_2_ receptor type 1/type 2
QoL: quality-of-life
RQLQ: rhinoconjunctivitis quality-of-life questionnaire
rTNSS: reflective total nasal symptom score
SD: standard deviation
SE: standard error
SAEs: serious adverse events
Th2 cells: T-helper type 2 cells
ULN: upper limit of normal
