# Supplementary material for: Efficacy and safety of setipiprant in seasonal allergic rhinitis: results from Phase 2 and Phase 3 randomized, double-blind, placebo- and active-referenced studies
Source: Allergy Asthma Clin Immunol. 2017 Apr 4;13:18. doi: 10.1186/s13223-017-0183-z (PMC5379543; doi:10.1186/s13223-017-0183-z)
Supplement: Supplementary file 1 — Additional file 1: Figure S1. Patient disposition in a) Phase 2 trial and b) Phase 3 trial. [file 13223_2017_183_MOESM1_ESM.docx]

**Supplementary Fig 1.** Patient disposition in a) Phase 2 trial and b) Phase 3 trial

**a)**


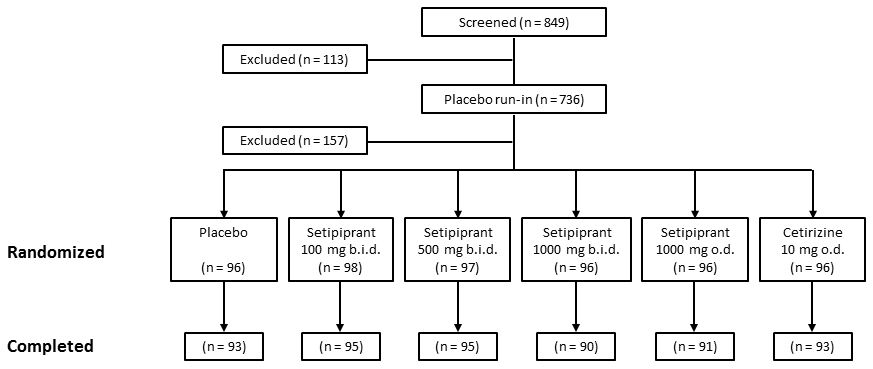


**b)**


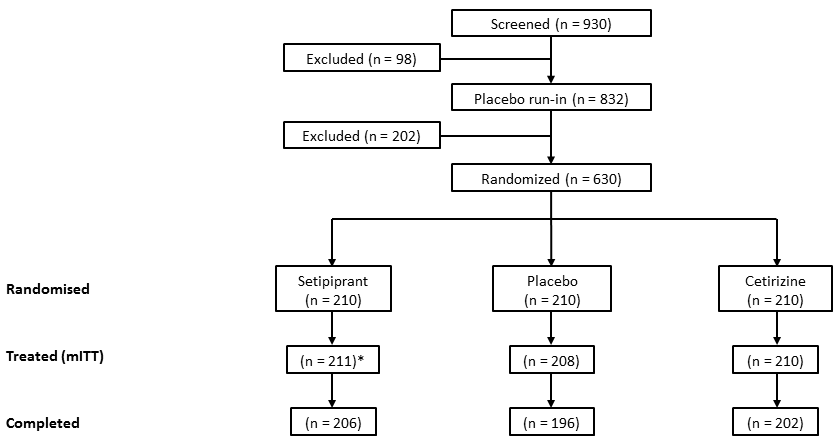


**Including one patient who was randomized to placebo, but received setipiprant during the double-blind treatment period.*
