# Supplementary material for: Efficacy and safety of setipiprant in seasonal allergic rhinitis: results from Phase 2 and Phase 3 randomized, double-blind, placebo- and active-referenced studies
Source: Allergy Asthma Clin Immunol. 2017 Apr 4;13:18. doi: 10.1186/s13223-017-0183-z (PMC5379543; doi:10.1186/s13223-017-0183-z)
Supplement: Supplementary file 2 — Additional file 2: Figure S2. Overall pollen counts by day prior to and during Phase 2 and Phase 3 trials. [file 13223_2017_183_MOESM2_ESM.docx]

**Supplementary Fig 2.** Overall pollen counts by day prior to and during Phase 2 and Phase 3 trials

**a) Phase 2: overall pollen counts**

**b) Phase 3**

*Data are mean ± SD across all seven study sites; negative horizontal axis numbers are days before treatment start; positive horizontal axis numbers are days during randomized treatment.*
