# Supplementary material for: Efficacy and safety of setipiprant in seasonal allergic rhinitis: results from Phase 2 and Phase 3 randomized, double-blind, placebo- and active-referenced studies
Source: Allergy Asthma Clin Immunol. 2017 Apr 4;13:18. doi: 10.1186/s13223-017-0183-z (PMC5379543; doi:10.1186/s13223-017-0183-z)
Supplement: Supplementary file 3 — Additional file 3: Table S1. Setipiprant trough plasma concentrations across dose groups at the end* of weeks 1 and 2 during Phase 2 and Phase 3 trials. [file 13223_2017_183_MOESM3_ESM.docx]

**Supplementary Table 1.** Setipiprant trough plasma concentrations across dose groups at the end* of Weeks 1 and 2 during Phase 2 and Phase 3 trials

|  | **PHASE 2 TRIAL** | | | | | | | | **PHASE 3 TRIAL** | |
| --- | --- | --- | --- | --- | --- | --- | --- | --- | --- | --- |
|  | **100 mg b.i.d.** | | **500 mg b.i.d.** | | **1000 mg b.i.d.** | | **1000 mg o.d.** | | **1000 mg b.i.d.** | |
|  | **Week 1** | **Week 2** | **Week 1** | **Week 2** | **Week 1** | **Week 2** | **Week 1** | **Week 2** | **Week 1** | **Week 2** |
| Arithmetic mean  (SD)  n | 285  (214)  84 | 290  (230)  84 | 1474  (1587)  77 | 1299  (1245)  77 | 2364  (2061)  69 | 2276  (2115)  69 | 2301  (2375)  74 | 1826  (2046)  74 | 1671  (2122)  173 | 1788  (2552)  173 |
| Geometric mean  (95% CI)  n | 230  (31, 1705)  76 | 248  (53, 1159)  77 | 832  (38, 18057)  75 | 672  (26, 17319)  76 | 1411  (74, 26756)  66 | 1222  (36, 41131)  67 | 1523  (82, 28265)  66 | 1145  (54, 24169)  63 | 982  (798, 1208)  163 | 885  (675, 1160)  160 |
| Median  (Range)  n | 251  (0–894)  84 | 241  (0–1310)  84 | 1110  (0–9810)  77 | 940  (0–7700)  77 | 1920  (0–8060)  69 | 1540  (0–13200)  69 | 1435  (0–10900)  74 | 1125  (0–9820)  74 | 1120  (0–16400)  173 | 1120  (0–22400)  173 |

**Pre-dose values based on samples taken before the morning dose on Days 7 and 14 of double-blind therapy.*
